# Growing from experience: an exploratory study of posttraumatic growth in adolescent refugees

**DOI:** 10.3402/ejpt.v7.28698

**Published:** 2016-02-12

**Authors:** Marieke Sleijpen, Joris Haagen, Trudy Mooren, Rolf J. Kleber

**Affiliations:** 1 Foundation Arq, Diemen, The Netherlands; 2 Department of Clinical & Health Psychology, Utrecht University, Utrecht, The Netherlands; 3 Foundation Centrum'45, Oegstgeest, The Netherlands

**Keywords:** asylum seekers, posttraumatic growth, posttraumatic stress, refugees, trauma, youth

## Abstract

**Objective:**

The aim of this study was to explore perceived posttraumatic growth (PTG) and its associations with potentially traumatic events (PTEs), dispositional optimism, perceived social support, posttraumatic stress disorder (PTSD) symptoms, and satisfaction with life (SWL) among adolescent refugees and asylum seekers.

**Method:**

A cross-sectional design was employed including 111 refugees, aged 12–17, that were recruited from asylum seeker centres throughout the Netherlands. Measurements included the revised Posttraumatic Growth Inventory for Children, Children's Impact of Event Scale, Multidimensional Scale of Perceived Social Support, The Life Orientation Test, and the Satisfaction with Life Scale.

**Results:**

Participants reported mean PTG scores (20.2) indicating an average response of some perceived change, while reporting high levels of PTSD symptoms (30.6). PTG and PTSD symptoms were not related with each other (*r*=0.07, *p=*0.50). PTG was positively associated with dispositional optimism (*r*=0.41, *p*<0.01) and social support (*r*=0.43, *p<*0.01). A hierarchical regression analysis demonstrated that dispositional optimism (β=0.33; *p*<0.05) and social support (β=0.27; *p*<0.05) positively predicted PTG, explaining 22% of the PTG variance above demographic variables and PTEs. PTG was also positively related with SWL (*r*=0.37, *p<*0.01).

**Conclusions:**

Perceived PTG and PTSD symptoms appear to be independent constructs, which co-occur in adolescent refugees and asylum seekers. The relationship between PTG and mental health remains inconclusive; PTG was positively related to SWL and not associated with PTSD symptoms. Longitudinal research is required to determine causality between PTG and mental health in this refugee population confronted with many traumatic experiences and challenging migration tasks.

Exposure to potentially traumatic events (PTEs) is a known risk factor for the development of mental health disturbances, while a growing body of empirical research also reveals that positive changes may take place after PTEs. This phenomenon of benefiting or growing psychologically from PTEs has been recognised throughout human history and is incorporated in the world's major religions (Splevins, Cohen, Bowley, & Joseph, 2010). Over the years, different names have been assigned to this phenomenon, but it is most frequently referred to as *benefit finding*, *stress-related growth*, or *posttraumatic growth* (PTG). Tedeschi and Calhoun (2004) defined PTG as a “positive psychological change experienced as a result of the struggle with highly challenging life circumstances” (p. 1). It manifests itself in improved interpersonal relationships, an increased sense of personal strength, positive changes in life priorities, and a richer existential and spiritual life (Tedeschi & Calhoun, 1996). Tedeschi and Calhoun suggested it is not the event itself but the struggle following the hardship that leads to PTG.

Although PTG has been identified in a variety of populations (children, adolescents, and adults) from different countries exposed to a range of PTEs (e.g., cancer, natural disasters, and bereavement), only a small number of studies have been conducted with adult refugees. To the authors’ knowledge, no quantitative studies have examined PTG among young refugees. PTG has been found to be present in adult refugees alongside negative consequences of trauma and ongoing difficulties, such as posttraumatic stress disorder (PTSD) symptoms (Hussain & Bhushan, 2011; Kroo & Nagy, 2011; Powell, Rosner, Butollo, Tedeschi, & Calhoun, 2003; Teodorescu et al., 2012).

There is contradictory evidence regarding the relationship between PTSD symptoms and PTG: Studies have found a significant positive relationship (e.g., Hall et al., 2010; Solomon & Dekel, 2007), a negative relationship (e.g., Frazier, Conlon, & Glaser, 2001), and no relationship at all (e.g., Widows, Jacobsen, Booth-Jones, & Fields, 2005). These conflicting findings on the relationship between PTSD and PTG might suggest a curvilinear relationship between PTG and PTSD; PTSD symptoms are first associated with an increase in PTG, but when the PTSD symptoms become more severe PTG decreases (e.g., Shakespeare-Finch & Lurie-Beck, 2014). Further, puzzling findings have been reported with regard to the relationship of PTG with more general adjustment outcomes, such as quality of life. Some studies reveal a positive relation between quality of life and PTG (e.g., Sim, Lee, Kim, & Kim, 2014), also in an adult refugee population (Teodorescu et al., 2012). However, a positive correlation of PTG with quality of life was not confirmed by a meta-analysis (Helgeson, Reynolds, & Tomich, 2006).

Given the obscurity of the PTG concept and the complex relationship between PTG and mental health, it is important to identify factors that are associated with PTG in survivors of traumatic events. In different populations, dispositional optimism (e.g., Feder et al., 2008; Kolokotroni, Anagnostopoulos, & Tsikkinis, 2014) and social support (e.g., Kroo & Nagy, 2011; Shand, Cowlishaw, Brooker, Burney, & Ricciardelli, 2014) were found to be core constructs predicting PTG. This is not surprising given the fact that feelings of greater closeness with others is an element of PTG, and the defining feature of dispositional optimism is having a positive outlook (Helgeson et al., 2006).

Controversy remains about the significance of PTG for psychological adjustment after PTEs, and more empirical evidence in different populations is highly needed. If PTG makes a difference in survivors’ lives by affecting levels of distress, quality of life, satisfaction with life (SWL), or other areas of mental health, it is relevant to study PTG in clinical research (Zoellner & Maercker, 2006). The proposed model of PTG by Tedeschi and Calhoun (2004) assumes that PTG is a positive and adaptive phenomenon, while the Janus-Face model by Zoellner and Maercker (2006) suggests that PTG not only has a constructive, self-transcending side, but also has a self-deceptive, illusory (non-beneficial) one. Although the concept of PTG seems universally acceptable, the PTG perspective was developed in the West and may represent a cultural bias emanating from individualistic societies. How moral qualities are valued might be culture-bound. For instance, autonomy is regarded highly important in an individualistic culture, whereas collectivist cultures place more emphasis on socially constructive behaviours (Splevins et al., 2010).

## Present study

During the last couple of years, the number of people seeking refugee status in Europe has been continuous and will continue to be driven, for example, by the wars in the Middle East (United Nations High Commissioner for Refugees, 2015). This has drawn researchers’ and mental health practitioners’ attention to the need to assess the psychological and social problems of this population. Studies investigating positive transformations among refugees have just started to take shape. The present study aims to expand this knowledge by exploring PTG among adolescent refugees and asylum seekers in the Netherlands, a specific group that has, thus far, received little academic attention (Fazel, Reed, Panter-Brick, & Stein, 2012; Sleijpen, Ter Heide, Mooren, Boeije, & Kleber, 2013).

Objectives of the study were the following: first, to identify socio-demographic as well as psychosocial variables associated with perceived PTG; and second, to explore the relationship between perceived PTG and mental health problems, specifically PTSD symptoms and dissatisfaction with life (defined in terms of global life dissatisfaction as a cognitive-judgemental process). In particular, the relationship between perceived PTG and the PTSD symptom cluster “intrusion” was examined because previous studies have shown that intrusive thoughts about PTEs were specifically related to PTG (Helgeson et al., 2006). In addition, we tested a curvilinear relation between PTG and PTSD symptoms.

As within other populations, we hypothesised that dispositional optimism (defined in terms of a person's positive expectations for the future) and perceived social support (defined in terms of subjectively assessed social support from family, friends, and significant others) account for additional variance in perceived PTG in adolescent refugees. On the basis of the view that higher levels of stress should stimulate more PTG (Calhoun & Tedeschi, 1998), we hypothesised that a larger number of experienced PTEs is associated with more perceived PTG. Furthermore, we hypothesised against the backdrop of (mixed) previous findings in other populations that perceived PTG would be associated negatively with PTSD symptoms (including intrusion symptoms) and positively with SWL in this population.

## Method

### Participants

One hundred twenty-four refugees and asylum seekers living in asylum seeker centres (ASCs) throughout the Netherlands were approached for participation. Six of them refused participation because they had other matters to attend (e.g., going to the doctor or playing soccer). Three refugees did not speak sufficient Dutch and a suitable interpreter was not available at the time. Another two refugees were not permitted to participate by their parents. Two refugees failed to complete the questionnaires because of fasting during Ramadan. The total response rate was 89.5%. Data from 111 participants were analysed: 54 boys (49%) and 57 girls (51%) and their age varied between 12 and 17 years (*M=*14.5, SD=1.8). The adolescents spent 3.4 years on average in the Netherlands; a third of the sample was granted asylum during this period (31%) (see Table 1 for demographics).

**Table 1:** Demographic data (*n*=107–111)

Variables	*N*	%
Gender		
Male	54	49
Female	57	51
Country of origin		
Iraq	24	22
Armenia	15	14
Syria	15	14
Afghanistan	13	12
Somalia	7	6
Other	37	33
Religious beliefs		
Christian	40	37
Muslim	64	59
Other	3	3
Atheist	1	1
Status		
Refugee	34	31
Asylum seeker	77	69
	*M*	SD
Age	14.5	1.8
Length of stay (years) in the Netherlands	3.4	2.7
No. of experienced PTEs	7.9	4.4
PTSD symptoms	30.6	14.4
Posttraumatic growth	20.2	5.8
Dispositional optimism	18.4	5.0
Social support	63.1	15.2
Satisfaction with life	18.2	7.8

### Measures

The collected demographic information included age, gender, country of origin, asylum status (whether or not having a residence permit), religious beliefs, and length of stay in the Netherlands. PTEs were measured by a 26-item questionnaire based on the UCLA PTSD Reaction Index DSM-IV (the part to screen for exposure to PTEs; Pynoos, Rodriguez, Steinberg, Stuber, & Frederick, 1998) and part I (trauma events) of the Harvard Trauma Questionnaire (HTQ; Mollica et al., 1992).

PTSD symptoms were measured with the Children's Revised Impact of Event Scale (CRIES-13; Smith, Perrin, Dyregrov, & Yule, 2003; Dutch version provided by Olff, 2005), a child-adapted version of the Impact of Event Scale (Horowitz, Wilner, & Alvarez, 1979). The questionnaire consists of 13 items answered on a 4-point Likert scale that indicates how frequently the participants experience symptoms (0=*not at all*, 1=*rarely*, 3=*sometimes*, and 5=*often*; range=0–65). The CRIES-13 includes three subscales: intrusion (four items), avoidance (four items), and arousal (five items). A cut-off score of 30 accurately classified 75–83% of the PTSD cases (Perrin, Meiser-Stedman, & Smith, 2005). Research on the psychometric properties of the questionnaire was done in different populations and countries and was rated *acceptable* to *good* (Giannopoulou et al., 2006; Perrin et al., 2005; Smith et al., 2003). Cronbach's alpha in the present study of the total scale was good (α=0.84).

Positive consequences of PTEs were assessed with the revised Posttraumatic Growth Inventory for Children (PTGI-C-R, Kilmer et al., 2009; Dutch version provided by Alisic, 2006). The PTGI-C-R was originally developed as an instrument for adult populations (Tedeschi & Calhoun, 1996). Kilmer et al. (2009) created an adapted version with fitted formulations for a younger population. The PTGI-C-R consists of 10 items (e.g., “I can now handle big problems better than I used to”; “I appreciate (enjoy) each day more than I used to”) based on five PTG domains (new possibilities, relating to others, personal strength, appreciation of life, and spiritual change) rated on a 4-point Likert scale (0=*no change*, 1=*a little change*, 2= *some change*, and 3=*a lot of change*). Higher scores represent increased PTG (range=0–30). There are no subscales. Cryder, Kilmer, Tedeschi, and Calhoun (2006) reported a Cronbach's alpha of 0.89, and Alisic, Van der Schoot, Van Ginkel, and Kleber (2008) reported 0.85 in a Dutch population, indicating good internal consistency. Cronbach's alpha in the present study was acceptable (α=0.73).

The Multidimensional Scale of Perceived Social Support (MSPSS; Zimet, Dahlem, Zimet, & Farley, 1988; Zimet, Powell, Farley, Werkman, & Berkoff, 1990; Dutch version provided by Pedersen, Spinder, Erdman, & Denollet, 2009) was used to measure the perceived level of social support from family, friends, and significant others. The questionnaire consists of 12 items; each item is answered on a 7-point Likert scale (1=*very strongly disagree* to 7=*very strongly agree*; range=12–84). The scale's psychometric properties are good, as determined by studies in different populations, for instance, adolescent populations (Bruwer, Emsley, Kidd, Lochner, & Seedat, 2008; Canty-Mitchell & Zimet, 2000). In addition, the MSPSS was cross-culturally tested as a reliable measuring instrument (Bagherian-Sararoudi, Hajian, Ehsan, Sarafraz, & Zimet, 2013; Bruwer et al., 2008). Cronbach's alpha in the current study was good (α=0.87).

The Life Orientation Test (LOT, Scheier & Carver, 1985; Dutch version provided by Vinck, Wels, Arickx, & Vinck, 1998) was used to measure dispositional optimism. This 12-item scale (with four filler items) assesses the extent to which an individual generally expects positive versus negative outcomes in life. Participants responded to statements on a 5-point Likert scale (0=*strongly disagree* to 4=*strongly agree*) of which four items had to be reversed prior scoring. Higher scores indicate higher optimism (range=0–32). The internal consistency and stability over time of the Dutch LOT were found to be sufficient in youth (Vinck et al., 1998). Cronbach's alpha in the current study was acceptable (α=0.67).

Global life satisfaction was assessed with the five items of the Satisfaction with Life Scale (SWLS; Diener, Emmons, Larsen, & Griffin, 1985; Dutch version provided by Arrindell, Meeuwesen, & Huyse, 1991), answered on a 7-point Likert scale (1=*strongly disagree* and 7=*strongly agree*; range=5–35). The Dutch version of this questionnaire reveals good reliability and high internal consistency (Arrindell et al., 1991). Furthermore, the SWLS appears to be cross-culturally applicable (Pavot & Diener, 1993). Cronbach's alpha in the current study was good (α=0.83).

### Procedures

We randomly contacted seven ASCs throughout the Netherlands, and they all gave permission for contacting asylum seekers living in their centres. Adolescents and their parents were orally informed about the purpose of the study and received an information letter. Both adolescents and their parents gave written permission for participation in the study. Inclusion criteria were aged between 12 and 17 and not born in the Netherlands. Mental retardation was an exclusion criterion as adolescents would not be able to understand the questions. Participants were recruited from July 2014 until March 2015. Participants filled out the questionnaires in private, and a professional psychologist and an interpreter were present, who could provide assistance, if necessary. To facilitate comprehension, we added the official meanings of difficult words and Dutch expressions to the booklets of questionnaires. Twenty-five participants received assistance from an interpreter. Participants received a debriefing letter and a small gift after completion. We placed the measure for SWL ahead of the measures for exposure, PTSD symptoms, and PTG to avoid a possible answer bias. This cross-sectional study was approved by the Medical Ethics Committee of the University Medical Centre Utrecht.

### Statistical analyses

#### Data preparation

Participants could have a maximum of 20% missing item scores per scale. This small percentage of missing values allowed for mean imputations per scale with negligible impact on the statistical analysis (Schafer & Graham, 2002; Van Buuren, 2012). Participants were excluded from the analyses if they had more than 20% missing items on a scale or missing demographic information. As a result, between zero and six participants—depending on the variable that was analysed—were excluded from correlational analyses, and eight (7%) participants were excluded from the hierarchical regression analysis. Two outliers were also excluded from the hierarchical regression analysis after inspection of the standardised residuals.

#### Correlations, *t*-tests, and chi-square test

Full Pearson's correlations were computed for the relationships between PTG, age, length of stay in the Netherlands, dispositional optimism, perceived social support, the number of experienced PTEs (events), PTSD symptom severity (including PTSD Intrusion subscale severity), and SWL. A partial Pearson's correlation was computed for each significant bivariate relationship with PTG to ascertain whether a significant relationship would remain after controlling for all other significant relationships. Independent *t*-tests were calculated to examine group differences between males and females as well as adolescents with a refugee or asylum status on key research variables (PTG, SWL, PTSD severity, length of stay in the Netherlands, dispositional optimism, perceived social support, number of traumatic events, and age). Both categorical variables were also compared with each other using Pearson's chi-square test.

#### Hierarchical regression

We conducted a hierarchical regression analysis with PTG as the dependent variable. The PTG predictors were added to the model in stepwise order. During the first step, we accounted for confounding effects from the following covariates: age, gender, asylum status, and length of stay in the Netherlands. The number of experienced PTEs was added in the second step to determine whether the amount of PTEs predicted PTG. During the third step, we added dispositional optimism and perceived social support to the model as the final predictors of PTG. All analyses were performed using SPSS.22.

## Results

### Sample characteristics

The adolescent refugee sample experienced eight PTEs on average. Most commonly, these concerned forced flight (100%), war zone presence (65%), exposure to combat action (55%), and the loss of a close family member or friend (55%). The least common PTEs were physical torture exposure (10%), divorced parents (9%), and sexual abuse (4%). The adolescents were asked to report the occurrence of 25 specific PTEs. These 25 PTEs were not comprehensive because 60% of the participants reported additional PTEs that were not included in the list (see Table 2).

**Table 2:** Prevalence rates of different types of experienced PTEs

Type	*N*	%
Forced flight	111	100
War zone	71	65
Loss of close family member or friend	61	55
Combat	60	55
Witness severe wounded	47	42
Bomb attack	40	36
Serious medical issues (self)	36	32
Murder of a family member	36	32
Physical violence or threatened outside primary care system	29	26
No food or water	28	26
No accommodation or housing	28	26
Serious physical illness or handicap parent or sibling	28	25
Forced separation family	26	24
Murdered friend, neighbour, or acquaintance	24	22
Loss of parent or sibling	23	21
Verbal abuse in primary care system	23	21
Sick without medical care	23	21
Serious psychological illness of parent or sibling	22	20
Severe traffic accident	17	16
Disaster	16	15
Prisoner or abducted	16	15
Physical violence in a primary care system	14	13
Physical torture	11	10
Divorce of parents	9	8
Sexual abuse	4	4
Other traumatic event	66	60

The total PTG scores of the adolescents ranged from 6 to 30, with a total mean score of 20.2 (SD=5.8), suggesting an average response of *some* perceived change (Kilmer et al., 2009). Exactly half of the sample (50%) scored above the cut-off score (≥30) of the CRIES-13, indicating a probable PTSD diagnosis (Perrin et al., 2005). The participants appeared (*M*=18.2, SD=7.8) “slightly dissatisfied with life” (Pavot & Diener, 1993). They reported lower optimism scores compared to American undergraduate women (*M*=18.4, SD=5.0 vs. *M*=21.4, SD=5.2, *t*(376)=5.2, *p*<0.0001) (Scheier & Carver, 1985) and scored lower on perceived social support compared to European adolescents (*M*=63.1, SD=15.2 vs. *M*=67.2, SD=9.6, *t*(183)=2.1, *p*=0.04) (Zimet et al., 1990).

The average PTG item score ranged from 1.7 (SD=1.2) to 2.3 (SD=0.97). The item with the lowest mean score was “I appreciate (enjoy) each day more than I used to.” The item with the highest mean PTG score was “I have new ideas about how I want things to be when I grow up.” 46% of all answers were recorded in the highest PTG response category, indicating *a lot of* perceived change after stress-related life events. Only 14% of all the PTG answers were recorded in the lowest response category, indicating no perceived change at all.

### Relationships between variables

Full Pearson's correlations (Table 3) demonstrated strong positive relationships between PTG and dispositional optimism (*r*=0.41, *p*<0.01) and perceived level of social support (*r*=0.43, *p*<0.01). These findings suggest that adolescents that perceive high levels of social support and have high levels of dispositional optimism are more likely to report high levels of PTG. Alternatively, adolescents that experience high levels of PTG are more likely to report high levels of perceived social support and dispositional optimism.

**Table 3:** Correlation matrix

		1	2	3	4	5	6	7	8	9
PTG	1	1								
Age	2	−0.15	1							
Length	3	−0.14	−0.03	1						
Optimism	4	0.41**	−0.04	−0.05	1					
Social	5	0.43**	−0.16	−0.12	0.47**	1				
PTEs	6	−0.02	0.18	0.07	−0.24*	−0.37**	1			
PTSD	7	0.07	0.12	−0.04	−0.23*	−0.03	0.37**	1		
PTSD-I	8	0.05	0.21*	−0.06	−0.28*	−0.07	0.42**	0.86**	1	
Satisfaction	9	0.37**	−0.20*	−0.41**	0.30**	0.40**	−0.28**	−0.23*	−0.25*	1

*Note*. PTG=posttraumatic growth score (PTGI-C-R). Age=age in years. Length=length of stay in the Netherlands in years. Optimism=dispositional optimism score (LOT). Social=perceived social support score (MSPSS). PTEs=number of potentially traumatic events. PTSD=posttraumatic stress disorder symptom score (CRIES-13). PTSD-I=PTSD Intrusion score (CRIES-13 subscale). Satisfaction=Satisfaction with life score [SWLS].

* *p<*0.05;

** *p<*0.01.

PTG was positively related to SWL (*r*=0.37, *p*<0.01). Besides PTG, dispositional optimism (*r*=0.30, *p*<0.01) and the perceived level of social support (*r*=0.40, *p*<0.01) were positively related to SWL. Adolescents with a refugee status reported higher life satisfaction than adolescents that had not (yet) obtained such a status (*M*=4.7, SD=1.6 vs. *M*=3.2, SD=1.3, *t*(105)=4.6, *p*<0.001). The age of the participant (*r*=−0.20, *p*<0.05), the length of stay in the Netherlands (*r*=−0.41, *p*<0.01), the number of experienced PTEs (*r*=−0.28, *p*<0.01), and PTSD symptom severity level (*r*=−0.23, *p*<0.05) were negatively related with satisfaction of life. Consequently, older adolescents without a refugee status that spend more years in the Netherlands and that experienced more PTSD symptoms and less PTG were more likely to report a lower SWL.

There were no differences in any research variables between males and females. Participants with and without a refugee status furthermore differed on length of stay in the Netherlands with a longer length of stay for participants without a status compared to those that successfully received a refugee status (*M*=4.1 years, SD=2.4 vs. *M*=1.7, SD=2.6, *t*(104)=−4.7, *p*<0.001). The chi-square test between gender and refugee or asylum status was also non-significant (*p=*0.57).

**Fig. 1:**
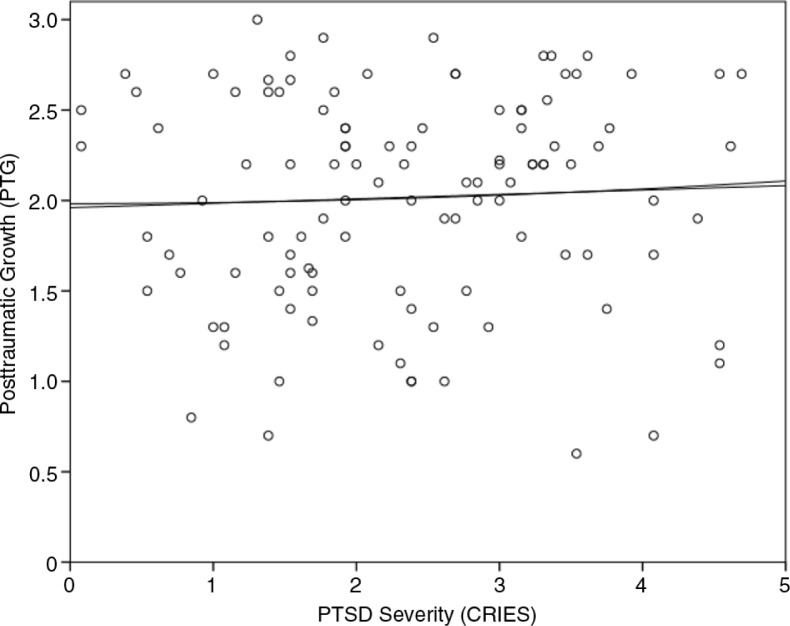
A scatterplot of PTSD severity and posttraumatic growth scores. *Note*. The lines depict the linear and curvilinear relation.

There was no relationship between the negative (PTSD) and positive (PTG) consequences of stressful life events (*r*=0.07, *p*=0.50), and also there was no relationship between the PTSD Intrusion subscale and PTG (*r*=0.05, *p*=0.64). Dispositional optimism was related to both PTG (*r*=0.41, *p*<0.01) and PTSD (*r=*−0.23, *p*<0.05). PTSD was negatively related to optimism, whereas PTG was positively related to optimism. PTSD was furthermore positively related to the total number of PTEs (*r*=0.37, *p*<0.01), unlike PTG (*r=*−0.02, *p*=0.84). This indicated that experiencing multiple PTEs increased PTSD severity levels but did not significantly affect the level of PTG among refugee adolescents. An inspection of the scatterplot between PTG and PTSD showed no evidence of a linear or curvilinear relationship between PTG and PTSD (Fig. 1). The positive significant relationship between PTG and dispositional optimism (*r*=0.19, *p*<0.05), perceived level of social support (*r*=0.19, *p*=0.05), and SWL (*r*=0.22, *p*<0.05) remained significant—albeit to a somewhat lesser extent—after controlling for all other significantly related PTG variables using partial Pearson's correlations.

### Hierarchical regression

The covariates (age, gender, asylum status, and length of stay in the Netherlands) predicted neither the PTG during the first step (*F*(4, 96)=1.08, *p*=0.37) nor the number of PTEs during the second step (*F*(5, 95)=0.86, *p*=0.51), although there appears to be a trend (*p*=0.06). The combined input of these predictors explained 4% of the PTG variance. In contrast, both dispositional optimism (β=0.33; *p*=0.002) and perceived social support (β=0.27; *p*=0.02) positively predicted PTG during the final step (*F*(7, 93)=4.79, *p*<0.001), explaining an additional 22% of the PTG variance and bringing the explained variance of the total model up to 27% (Table 4). In sum, after controlling for socio-demographic data, increased levels of dispositional optimism and perceived social support were related to increased levels of PTG.

**Table 4:** Hierarchical multiple regression analysis predicting posttraumatic growth

Predictor		B (SE)	CI (B)	β	Δ*R*^2^
Step 1					0.04
	Age	−0.04 (0.03)	−0.10, 0.02	−0.13	
	Gender	−0.04 (0.10)	−0.23, 0.16	−0.03	
	Status	−0.03 (0.11)	−0.26, 0.20	−0.03	
	Length	−0.01 (0.02)	−0.05, 0.03	−0.07	
Step 2					0.01
	PTEs	0.02 (0.01)	0.00, 0.05	0.19	
Step 3					0.22***
	Optimism	0.27 (0.09)**	0.10, 0.45	0.31**	
	Social support	0.13 (0.05)*	0.03, 0.22	0.29**	
Total *R*^2^	0.27***				
*n*	101				

*Note*. PTEs=number of potentially traumatic events. (Gender and status were dummy coded.) Gender: 0=male, 1=female. Status: 0=asylum seeker status, 1=refugee status. Length=length of stay in the Netherlands in years. CI=confidence interval (95%). B=unstandardized regression coefficient.

* *p<*0.05;

** *p<*0.01;

*** *p<*0.001.

## Discussion

The main objective of this study was to examine perceived PTG in refugee adolescents and its relationship with social support, dispositional optimism, PTSD symptoms, and SWL. To the authors’ knowledge, this is the first quantitative investigation of PTG in this vulnerable population. Participants represented a diverse group of asylum seekers and refugees who lived in ASCs throughout the Netherlands and had experienced a range of PTEs.

Adolescent refugees and asylum seekers perceived PTG. The PTG total score in this sample approximately equals the score of children impacted by Hurricane Katrina [a total PTG mean score of 20.0 (SD=6.5), 12 months post-hurricane; Kilmer et al., 2009] and is much higher than the average outcome of a representative sample of the Dutch population of youngsters [a total PTG mean score of 11.7 (SD=7.7); Alisic et al., 2008]. This finding implies that adolescent refugees and asylum seekers experience PTG in response to multiple PTEs and ongoing post-migration difficulties. It lends support to theoretical assertions that traumatic experiences may lead to the onset of a search for meaning and a fundamental reconfiguration of a person's life goals (Tedeschi & Calhoun, 2004).

We did not find a (curvilinear) relationship between PTSD symptoms and perceived PTG as well as between intrusion symptoms and perceived PTG. These findings confirm the notion that PTG and PTSD are two independent dimensions instead of extremes on a single continuum (Alisic et al., 2008; Linley & Joseph, 2004). In relation to SWL, this study did find a positive relationship with PTG. Although any inference of causality is impossible given the cross-sectional nature of the study, it might be suggested that high levels of perceived PTG can lead to life satisfaction.

Overall, the relationship between PTG and mental health in adolescent refugees remains unclear: PTG was positively related to SWL and unrelated with PTSD symptoms. It is debatable whether PTG is an outcome or a coping process that can be unsuccessful or ongoing and, consequently, not necessarily related to better mental health. Researchers have argued that at an early stage of trauma, PTG reflects a coping process rather than an outcome (e.g., Helgeson et al., 2006). Despite the fact that many refugees in this study have stayed for some time in the Netherlands (on average more than 3 years), their current situation in the ASCs and their worries about the asylum procedure are still highly stressful (Laban, Gernaat, Komproe, Van der Tweel, & De Jong, 2005). Possibly, a safe environment is needed before PTG also influences distress levels. More awareness of differences in individual stability of PTG is needed (Jayawickreme & Blackie, 2014).

Dispositional optimism and social support were positively associated with perceived PTG in adolescent refugees; they accounted for 22% of the variation. This result is consistent with previous studies in other populations and coherent with the theoretical conception of PTG. Accordingly, this expected finding adds to the validity of measuring PTG in non-western populations. It is reasonable to assume that more optimistic adolescents are better able to positively reframe their experiences and derive benefits from them (Fredrickson, 2001), and those who have more social support perceive greater PTG. The availability of social support can facilitate a process of change by evoking communication about traumatic experiences (Sutton, Robbins, Senior, & Gordon, 2006; Tedeschi & Calhoun, 2004). Using words may help adolescent refugees to process what had happened and lead to increased understanding. However, due to the cross-sectional nature of this study, causality cannot be assumed.

Remarkably, the present study did not find a significant relationship between the number of PTEs and PTG. (Only a trend towards the effect of PTEs on PTG became visible in the hierarchical regression.) Possibly, little differentiation could be made because many participants experienced multiple PTEs, whereas degrees of severity of the PTEs were ignored. Also no significant relations emerged between sample socio-demographic characteristics and PTG. Social-demographic variables, such as length of stay in the Netherlands and not having a permit for residency, however, did have a negative relationship with SWL. The longer asylum seekers stayed in the Netherlands, the lower their SWL in general. This can be explained by post-migration stress, such as the long asylum procedure, which promotes a continuous fear of deportation, uncertainty about the future, and difficult living conditions (Laban et al., 2005; Robjant, Hassan, & Katona, 2009).

The present study also indicated that PTG is not merely a “western” phenomenon (e.g., Kroo & Nagy, 2011) but a relevant cross-cultural concept. Key elements measured with the psychometric tool (PTGI-C-R), such as a richer spiritual life and positive changes in self-perception and future thoughts, correspond to the findings of a qualitative study of PTG among refugee minors (Sutton et al., 2006). Nevertheless, it might be premature to equate growth directly with something positive. A qualitative study among adolescent refugees showed that all youngster perceived growth; they had become stronger and more independent through the hardship they endured, but they did not perceive this as particularly positively because they had lost their childhood (Sleijpen, Mooren, Kleber, & Boeije, 2015). These young refugees appeared to have become wiser, but sadder individuals.

## Limitations

This study has several limitations. First, the findings rely on self-reports, whereas clinical interviews are seen as more valid in measuring psychopathology. This study assessed self-reported PTG, meaning that it has investigated whether participants believe that they have psychologically grown following PTEs. This might not reflect real transformation, but the ability to find a bright side in otherwise devastating circumstances (McFarland & Alvaro, 2000). Moreover, the assessment of PTG, as in the PTGI-C-R, is retrospective; participants were asked to estimate if and how much they have changed since the event(s) in different domains. It measures perceptions of change rather than actual pre- to post-trauma changes (Jayawickreme & Blackie, 2014). Nevertheless, if these self-perceptions of perceived growth are to some extent an illusion, but still help individuals to feel better, they can teach us something about what promotes trauma recovery (Jayawickreme & Blackie, 2014).

Another limitation of this study is its cross-sectional nature that renders it impossible to determine causality. Longitudinal studies are needed to untangle the relationships. Although we used questionnaires validated for different ethnic groups, it is possible that they did not accurately reflect the experiences in the different cultures (Hall et al., 2014; Kroo & Nagy, 2011; Splevins et al., 2010). Some of the PTGI-C-R's items, for instance, “I have new ideas about how I want things to be when I grow up,” could have another meaning for our participants because they had to leave their lives behind and adjust to the conditions of the host country. Processes underlying adolescent's responses to the questions could be changeable within time. Future research would benefit from a use of longitudinal mixed method designs to fully explore post-trauma trajectories in a culture-sensitive context. Ideally, study designs with baseline data collected prior to the PTEs are needed to study genuine positive transformations (Jayawickreme & Blackie, 2014), but this is rather impossible in the case of research on refugee populations.

## Conclusion

Adolescent refugees and asylum seekers living in ASCs throughout the Netherlands were exposed to multiple PTEs. In response, they reported high levels of PTSD symptoms as well as PTG. PTG was unrelated to PTSD symptoms and positively related to SWL. Social support and dispositional optimism were positively related to PTG. Greater understanding of processes that facilitate growth experiences in refugee adolescents is highly relevant, in particular because of the severe nature of their frequent traumatic experiences and the challenging task to adapt to the many changes they face.
